# Glycolipid metabolic status of overweight/obese adolescents aged 9- to 15-year-old and the BMI-SDS/BMI cut-off value of predicting dyslipidemiain boys, Shanghai, China: a cross-sectional study

**DOI:** 10.1186/1476-511X-12-129

**Published:** 2013-08-28

**Authors:** Chun-dan Gong, Qiao-ling Wu, Zheng Chen, Dan Zhang, Zheng-yan Zhao, Yong-mei Peng

**Affiliations:** 1 Department of Child Health Care, Children’s Hospital of Fudan University, Shanghai 201102, China; 2 Children’s Hospital of Medical College of Zhejiang University, Zhejiang 310003, China

**Keywords:** Adolescents, Children, Lipid metabolism, Obesity, Overweight, BMI-SDS, China

## Abstract

**Background:**

The prevalence of adolescents’ obesity and overweight has dramatically elevated in China. Obese children were likely to insulin resistance and dyslipidemia, which are risk factors of cardiovascular diseases. However there was no cut-off point of anthropometric values to predict the risk factors in Chinese adolescents. The present study was to investigate glycolipid metabolism status of adolescents in Shanghai and to explore the correlations between body mass index standard deviation score (BMI-SDS) and metabolic indices, determine the best cut-off value of BMI-SDS to predict dyslipidemia.

**Methods:**

Fifteen schools in Shanghai’s two districts were chosen by cluster sampling and primary screening was done in children aged 9-15 years old. After screening of bodyweight and height, overweight and obese adolescents and age-matched children with normal body weight were randomly recruited in the study. Anthropometric measurements, biochemical measurements of glycolipid profiles were done. SPSS19.0 was used to analyze the data. Receiver operating characteristic (ROC) curves were made and the best cut-off values of BMI-SDS to predict dyslipidemia were determined while the Youden indices were maximum.

**Results:**

Five hundred and thirty-eight adolescents were enrolled in this research, among which 283 have normal bodyweight, 115 were overweight and 140 were obese. No significant differences of the ages among 3 groups were found. There were significant differences of WC-SDS (p<0.001), triacylglycerol (p<0.05), high and low density lipoprotein cholesterol (p<0.01), fasting insulin (p<0.01) and C-peptide (p<0.001) among 3 groups. Significant difference of fasting glucose was only found between normal weight and overweight group. Significant difference of total cholesterol was found between obese and normal weight group. There was no significant difference of glycated hemoglobin among 3 groups. The same tendency was found in boys but not in girls. Only HDL-C reduced and TG increased while BMI elevated in girls. The best cut-off value of BMI-SDS was 1.22 to predict dyslipidemia in boys. The BMI cut-off was 21.67 in boys.

**Conclusion:**

Overweight and obese youths had reduced insulin sensitivity and high prevalence of dyslipidemia.When BMI-SDS elevated up to 1.22 and BMI was higher than 21.67 in boys, dyslipidemia may happen.

## Introduction

Overweight and obesity in children and adolescents is a public health problem worldwide. The prevalence of overweight and obesity in China youth has increased to 13.1% and 7.5% in 2006–2010 from 1.8% and 0.4% in 1981- 1985 [1], respectively. Obese children were several times more likely than normal-weight children to have hypertension, high insulin, dyslipidemia and high uric acid [2]. Increased total cholesterol (TC), low-density lipoprotein cholesterol (LDL-C), triglyceride (TG), and decreased high density lipoprotein cholesterol (HDL-C) are risk factors of cardiovascular diseases (CVD), which are the leading cause of mortality [3-5]. Studies have showed that about one-third children had elevated total cholesterol [6,7]. One study based on a large sample showed that the prevalence of metabolic syndrome in obese adolescents was 27.6%, which was pretty high [8].

As far back to 1980’s, mass screening of blood cholesterol as routine health care of children was discussed in several industrialized countries [9-11] and some countries have established screening programs [12]. The American Academy of Pediatrics (AAP) reemphasized the need for prevention of cardiovascular diseases and advocated to screening lipid profile in overweight children regardless of family history or other risk factors [13]. But as to developing countries, mass screen may be heavy economic burdens. So it became very important to know in which case blood test should be done.

Some researches about the cut-off point to predict CVD risk factors were done. Skinner, et al. found that TC increased significantly at the 80^th^ percentile while glycohemoglobin significantly increased at the 99^th^ percentile [14]. The results showed that TC had already elevated in some healthy weight adolescents. Maryam Barzin, et al. determined that the odds ratio for BMI z-score to predict metabolic syndrome was 2.6 in 6-12-year-olds and the optimal cut-off values for BMI were 16.5 kg/m^2^ for boys and 16.3 kg/m^2^ for girls [15].

To update, there was no study aiming to determine the cut-off value to predict the risk factors of CVD in Chinese adolescents. Hence our study intended to illustrate the characteristics of parameters of glycolipid metabolism in obese and overweight adolescents and analyze relevant factors thus to find the best out-off value of BMI-SDS to predict dyslipidemain Chinese youth.

## Material and methods

### Subjects

Fourteen elementary and junior high schools in Shanghai’s two districts were chosen by using cluster sampling. Questionnaires investigating body weight and height and physical activities were distributed to all the 9 to 15 years old adolescents. The subjects’ body weight and height were measured by trained health care teachers. Totally 10,423 adolescents were screened, among which 1,320(12.66%) were overweight, 699(6.70%) were obese and 8404 have normal weight. Depending on BMI, subjects were divided into normal weight group and high BMI group according to the Chinese BMI-for-age/gender reference [16]. In high-BMI group, 1,613 adolescents were randomly invited to have a physical examination by distributing informed consent forms. In normal weight group, 1688 adolescents were invited. The inclusion criteria conclude that the subjects should be single birth, without congenital diseases, chromosome abnormality, hereditary metabolic diseases and abnormal skeletal development. Five hundred and twenty-six consent forms of high BMI group and 824 of normal BMI group were retrieved. The subjects were contacted by telephone and were told the dos and don’ts before examination. The physical examinations were performed in batches. Finally 255 overweight and obese students and 283 normal weight students were included in this research. Signed informed consent was obtained from each child’s parent or guardian. Ethical approval was obtained from ethics committee of Children’s Hospital of Fudan University.

### Diagnose of obesity and overweight

Obesity was defined on the basis of BMI above the 95th percentile for age and gender on the BMI percentile charts for Chinese children and adolescents and overweight above the 85th percentile [17].

### Anthropometric measurements

Team members of this study were trained before research. Body weight and height were obtained using Seca stadiometer and electronic scale, with the participants wearing light underwear and barefoot. Waist circumference was measured at the narrowest point between the lower border of the rib cage and the iliac crest. Physical examination was performed to evaluate the participants’ health status. Non alcoholic fatty liver disease (NAFLD) was identified by ultrasonography and recorded as absent, mild, moderate and severe [18]. The examination was performed by the same radiologist using Premier-Siemens equipment.

### BMI-SDS and WC-SDS calculation

Body mass index (BMI) was calculated according to the equation: BMI = body weight (Kg)/ height^2^ (m^2^). As BMI and WC change with age and sex, BMI standard deviation score (BMI-SDS) and WC standard deviation score (WC-SDS)were calculated by LMS (L(lambda), M (mu), S (sigma)) method [19] for each one according to the references of BMI and WC distribution of Chinese children and adolescents [16,20].

$$\mathrm{BMI}-\mathrm{SDS}=\frac{{\left[\frac{\mathrm{BMI}}{M}\right]}^{L}‒1}{\mathrm{LS}}$$

$$\mathrm{WC}-\mathrm{SDS}=\frac{{\left[\frac{\mathrm{WC}}{M}\right]}^{L}‒1}{\mathrm{LS}}$$

### Biochemical measurements

Blood samples were taken from the participants after at least 8-hour fast. Total cholesterol, triglyceride, high density lipoprotein cholesterol (HDL-C), low density lipoprotein cholesterol (LDL-C), apolipoprotein A1, Apolipoprotein B, glucose, glycatedhemoglobin ratio, serum lipoprotein a, insulin, peptide C were tested by standard methods using a Hitachi 7180 analyzer (Hitachi High Technologies Corp, Tokyo, Japan). The coefficients of variation (CV) of intra-assay were less than 8% and inter-assay less than 10% for all the items. Electrochemical Luminescence was used to measure insulin and peptide C by Roche E601 analyzer. The CV of intra-assay and intermediate precision were ≤1.50% and ≤4.90% for insulin, ≤4.60% and ≤5.00% for peptide C.

### Definition of lipid and glucose abnormalities

According to the IDF criteria hypertriglyceridemia is defined as ≥150 mg/dL (1.70 mmol/L) for triacylglycerol, low HDL-C < 40 mg/dL (1.03 mmol/L) and hyperglycaemia as fasting glucose ≥ 5.6 0 mmol/L [21]. The cut points for elevated concentration are >200 mg/dL (5.18 mmol/L) for cholesterol and >130 mg/dL (3.37 mmol/L) for LDL-C [13].

### Statistical analysis

SSPS 19.0 was used to perform statistical analysis. For normally-distributed variables, mean ± standard deviation of data was given. For non-normally distributed data, median, interquartile range was given. Student-Newman-Keuls test was used to compare the means among groups while data met the criteria. Otherwise medians were compared by Kruskal-Wallis Htest. Chi-square test was used to compare the prevalence of fatty liver, high TC and other dyslipidemia among different groups. Simple correlations were done to calculate the correlation indices between BMI-SDS percentiles and the variables. Receiver operating characteristic (ROC) curves of BMI-SDS to predict hypertriglyceridemia and low HDL-C and high LDL-C were made. The best cut-off values were determined while the Youden index was maximum [22].

Youdenindex=sensitivity+specificity‒1.

## Results

Five hundred and thirty-eight adolescents participated in this research, among which 283 had normal body weight, 115 were overweight and 140 were obese. Table 1 shows the characteristics of the subjects. The means of BMI, BMI-SDS, WC, WC-SDS, TC, TG, LDL-C, insulin, HOMA-IR and C-peptide of obese subjects were significantly higher and HDL-C significantly lower than those of normal weight and overweight subjects. The level of glucose of normal weight children were lower than which in overweight group (p = 0.014). No statistically differences of hemoglobin A1c were found among the three groups.

**Table 1:** **Clinical and biochemical parameters of normal weight**, **overweight and obese adolescents**

**Variables**	**<85**^**th **^**percentile**	**85**^**th**^**-94**^**th **^**percentile**	≥**95**^**th **^**percentile**
All	283	115	140
Male	156	81	104
Female	127	34	36
Age(yr)	12.00(11.00,13.00)	12.00(11.00,13.00)	12.00(10.00,13.00)
BMI(kg/m^2^)	18.22 ± 2.06^***^	22.84 ± 1.42^¶¶¶^	27.57 ± 3.62^§§§^
BMI-SDS	0.16(−0.52,0.57) ^***^	1.50 ± 0.28^¶¶¶^	2.46 ± 0.55^§§§^
WC(cm)	64.62 ± 5.92^***^	76.32 ± 5.21^¶¶¶^	86.84 ± 8.19^§§§^
WC-SDS	0.15 ± 0.72^***^	1.33 ± 0.33^¶¶¶^	1.97 ± 0.36^§§§^
TC(mmol/L)	4.21 ± 0.67	4.27 ± 0.69	4.44 ± 0.76^§§^
TG(mmol/L)	0.77(0.60,0.97)^*^	0.81(0.63,1.26)^¶¶¶^	1.02(0.83,1.48)^§§§^
HDL-C(mmol/L)	1.55 ± 0.30^***^	1.41 ± 0.24^¶¶^	1.32 ± 0.23^§§§^
LDL-C(mmol/L)	2.28 ± 0.56^**^	2.46 ± 0.59^¶¶^	2.67 ± 0.66^§§§^
Glucose(mmol/L)	4.83 ± 0.43^*^	4.94 ± 0.42	4.90 ± 0.48
HbA_1C_%(mg/L)	4.70(4.55,4.89)	4.70(4.60,4.90)	4.73(4.60,4.92)
Insulin(μIU/ml)	5.57(4.01,7.81)^**^	6.47(5.04,9.88)^¶¶¶^	9.83(5.97,15.98)^§§§^
HOMA-IR	1.19(0.86,1.64)^**^	1.51(1.07,2.12)^¶¶¶^	2.12(1.25,3.24)^§§§^
C-P(ng/ml)	1.17(0.74,1.69)^***^	1.51(1.07,2.12)^¶¶¶^	2.11(1.52,3.05)^§§§^

*BMI* Body mass index, *BMI-SDS* Body mass index standard deviation score, *WC* Waist circumference, *WC-SDS* Waist circumference standard deviation score, *TC* Total cholesterol, *TG* Triglyceride, *HDL-C* High density lipoprotein cholesterol, *LDL-C* Low density lipoprotein cholesterol, *HbA*_*1C*_*%* Hemoglobin A1c, *C-P* Peptide C, *HOMA-IR* Homeostasis model assessment of insulin resistance, Mean or median values were significantly different, normal weight vs. overweight: ^*^p < 0.05, ^**^p < 0.01,^***^p < 0.001.

Mean or median values were significantly different, overweight vs. obese p < 0.05,^¶¶^ p < 0.01,^¶¶¶^ p < 0.001 Mean or median values were significantly different, normal weight vs. obese: ^§^p < 0.05, ^§§^p < 0.01,^§§§^p < 0.001.

The prevalence of dyslipidemia and NAFLD among three groups was shown in Table 2. The ratios of NAFLD and hypertriglyceridemia were significantly lower in normal weight children than in overweight children. But no statistically differences were found in the ratios of other dyslipidemia among these two groups. Except the ratio of hyperglycaemia, the prevalence of NAFLD and dyslipidemia in obese children were significantly higher than those in normal weight children.

**Table 2:** Prevalence of CVD risk factors among normal weight, overweight and obese children

	**Total**	**<85**^**th**^	**85**^**th**^-**95**^**th**^	≥**95**^**th**^
**Abnormalities**		**percentile**	**percentile**	**percentile**
	**N = 538**	**N=283**	**N=115**	**N=140**
NAFLD (n/%)	91 (16.91)	3 (1.06) ^***^	15 (13.04)^¶¶¶^	73 (52.14) ^§§§^
Hypertriglyceridemia (n/%)	28 (5.20)	3 (1.06) ^***^	10 (8.70)	15 (10.71) ^§§§^
Hyperglycaemia (n/%)	26 (4.83)	10 (3.53)	5 (4.35)	11 (7.86)
High TC (n/%)	43 (7.99)	13 (4.59)	10 (8.70)	20 (14.29)^§§§^
Low HDL-C (n/%)	16 (2.97)	3 (1.06)	4 (3.48)	9 (6.43)^§§^
High LDL-C (n/%)	33 (6.13)	7 (2.47)	9 (7.83)	17 (12.14)^§§§^

*TC*, total cholesterol; *HDL-C*, high density lipoprotein cholesterol; *LDL-C*, low density lipoprotein cholesterol.

Ratios were significantly different, normal weight vs. overweight: ^***^p < 0.001.

Ratios were significantly different, overweight vs. obese: ^¶¶¶^p < 0.001.

Ratios were significantly different, normal weight vs. obese: ^§§^p < 0.01,^§§§^p < 0.001.

**Figure 1:**
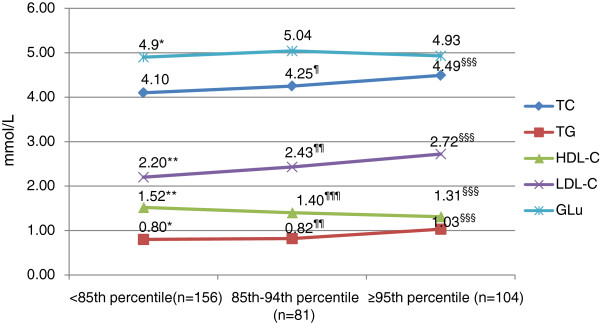
**Glycolipid indices in boys with different BMI percentiles.** TC, total cholesterol; TG, triglyceride; HDL-C, high density lipoprotein cholesterol; LDL-C, low density lipoprotein cholesterol; Glu, glucose. Difference between normal weight and overweight: *p<0.05, **p<0.005. Difference between overweight and obese: ^¶^p<0.05, ^¶¶^p<0.005, ^¶¶¶^p<0.001. Difference between normal weight and obese: ^§§§^p<0.001.

**Figure 2:**
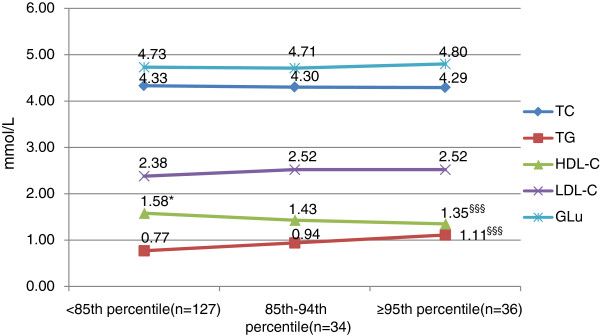
**Glycolipid indices in girls with different BMI percentiles.** TC, total cholesterol; TG, triglyceride; HDL-C, high density lipoprotein cholesterol; LDL-C, low density lipoprotein cholesterol; Glu, glucose. Difference between normal weight and overweight: *p<0.05. Difference between normal weight and obese: ^§§§^p<0.001.

**Figure 3:**
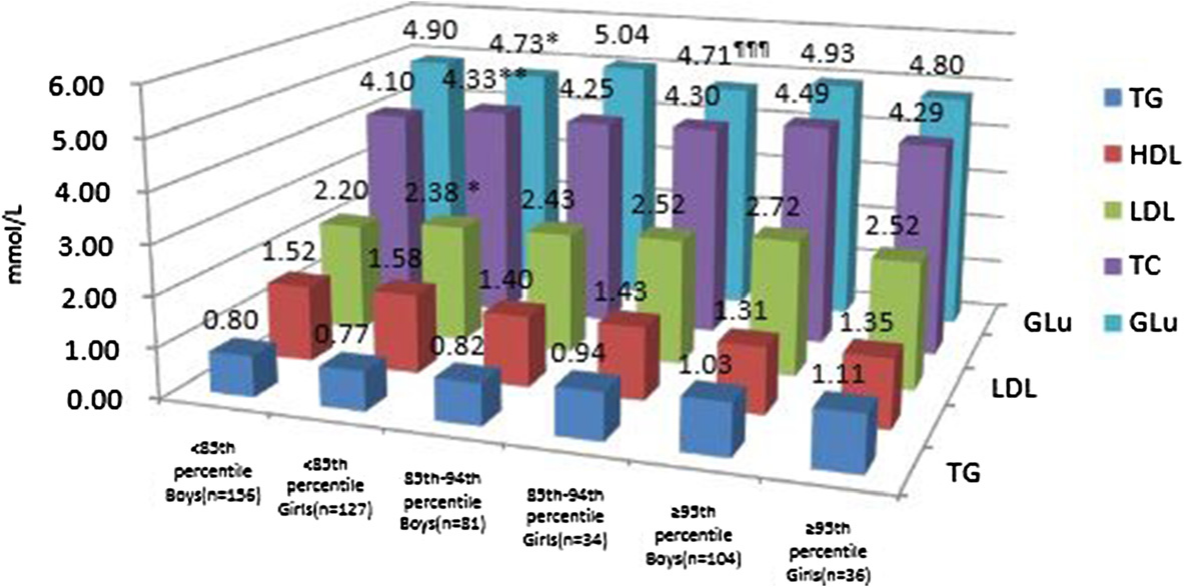
**Glycolipid indices in boys with defferent BMI percentiles.** Difference between boys and girls with BMI <85^th^ percentile: *p<0.05, **p<0.01. Difference between boys and girls with BMI between <85^th^ and <94^th^ percentile: ^¶¶¶^p<0.01.

The concentrations of lipids and glucose in boys were shown in Figure 1. Briefly, the mean levels of TC, TG, LDL-C were elevated while BMI increased, with HDL-C reduced. This tendency was not found in the levels of glucose. The indices of girls were shown in Figure 2. The mean concentration of HDL-C was significantly lower in obese and overweight subjects than in normal weight ones. And the mean level of TG in obese ones was significantly higher than normal ones. No significances were found in other indices among different BMI groups. Differences between genders were also compared and shown in Figure 3. In normal weight groups, the mean levels of LDL-C and TC were significantly higher in girls than those in boys. The glucose levels in girls were significantly lower than in boys in normal and overweight groups.

The correlations between BMI percentiles and the glycolipid profiles were shown in Table 3. BMI percentiles were positively correlated with TG, LDL-C, HOMA-IR, insulin and NAFLD and conversely correlated with HDL-C. Correlation was found between BMI-SDS percentiles and TC,LDL-C in boys but not in girls.

**Table 3:** Spearman correlation coefficients between BMI percentiles and laboratory parameters

	**Total**		**Boys**		**Girls**	
	**R**	**P**	**R**	**P**	**R**	**P**
TC	0.09	0.036	0.21	<0.001	−0.09	0.211
TG	0.32	<0.001	0.36	<0.001	0.23	0.001
HDL-C	−0.35	<0.001	−0.33	<0.001	−0.34	<0.001
LDL-C	0.24	<0.001	0.33	<0.001	0.09	0.237
HOMA-IR	0.31	<0.001	0.34	<0.001	0.21	0.003
Insulin	0.32	<0.001	0.36	<0.001	0.25	0.001

*TC* Total cholesterol, *TG* Triglyceride, *HDL-C* High density lipoprotein cholesterol, *LDL-C* Low density lipoprotein cholesterol; homeostasis model assessment of insulin resistance.

The ROC curve reflecting high level of TC, TG, LDL-C and low level of HDL-C from BMI-SDS in boys was shown in Table 4. The cutoff points for predicting increased TC, TG and LDL-C were 1.29 and 1.27, which were close to each other. But the value to predict reduced HDL-C was 2.19, relatively higher than others.

**Table 4:** AUCs and cutoff points of BMI-SDS to predict abnormal lipid profiles in boys

	**AUC**	**95% CI**	**Cutoff**	**Sensitivity**	**Specificity**
TC	0.727	0.642-0.812^***^	1.29	87.5	58.0
TG	0.774	0.695-0.853^***^	1.27	90.9	57.1
HDL-C	0.809	0.695-0.924^***^	2.19	69.2	84.8
LDL-C	0.756	0.686-0.827^***^	1.29	90.5	82.7

*BMI-SDS* Body mass index standard deviation score, *TC* Total cholesterol, *TG* Triglyceride, *HDL-C* High density lipoprotein cholesterol, *LDL-C* Low density lipoprotein cholesterol, *CI* Confidence interval, *AUC* Area under the ROC curve. *** P < 0.001.

**Figure 4:**
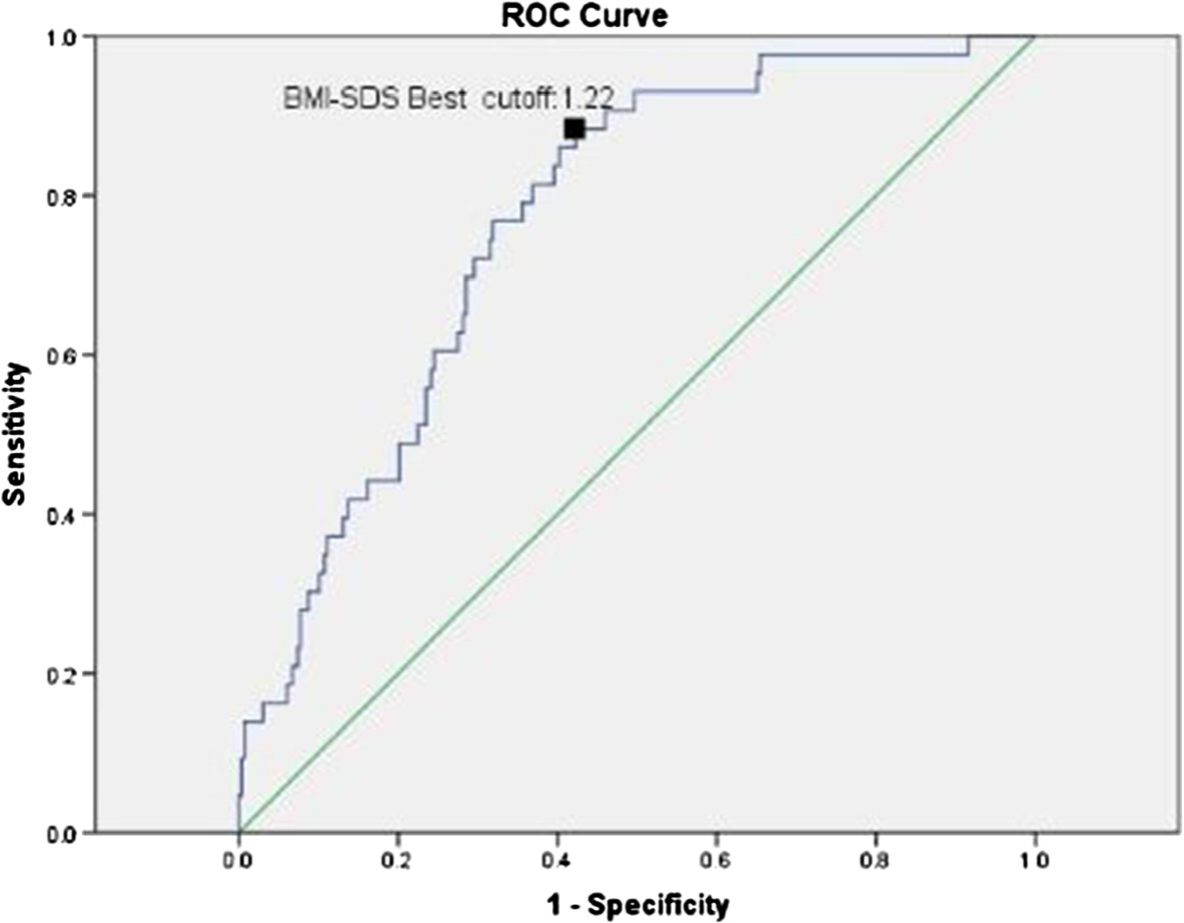
Reciever operating characteristic curve of BMI SDS reflecting dyslipidemia in boys.

**Figure 5:**
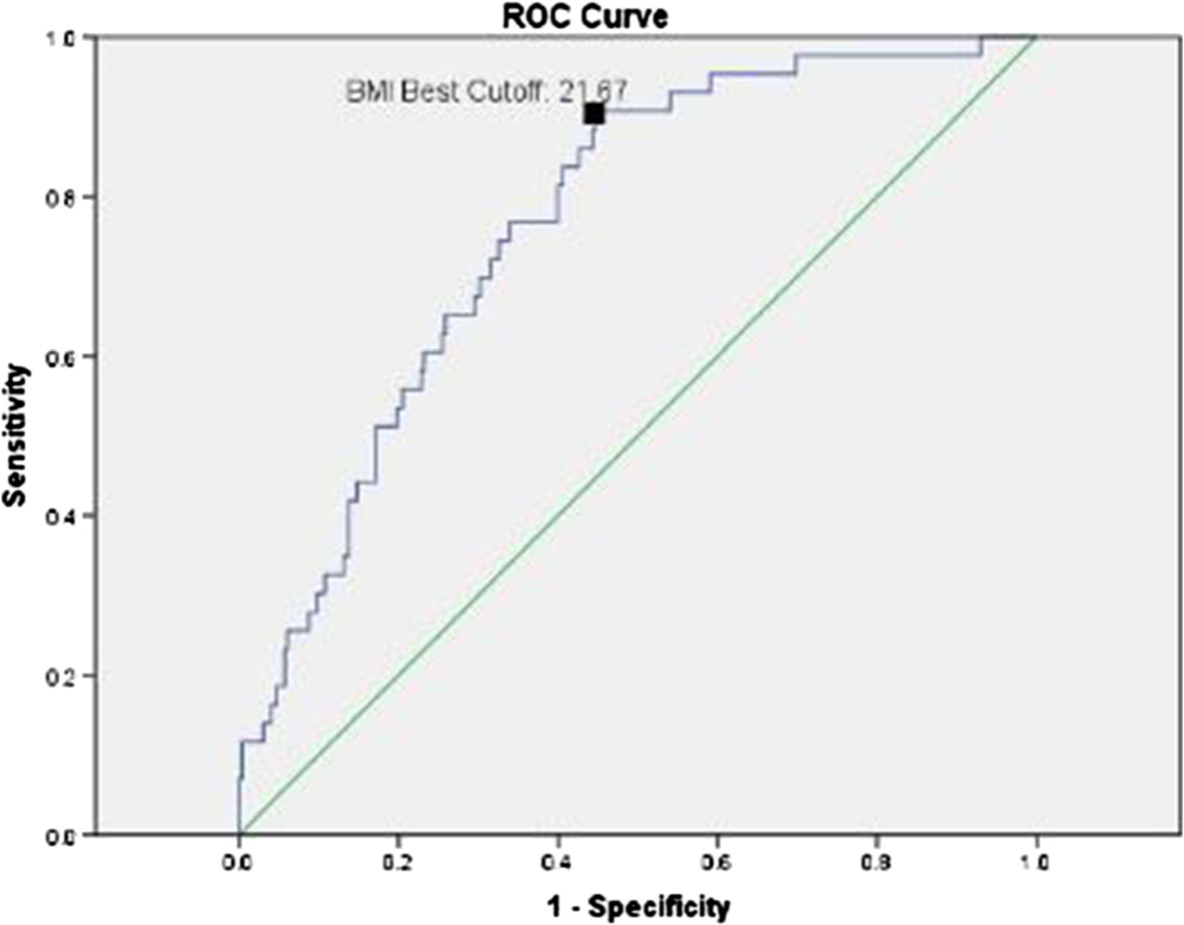
Reciever operating characteristic curve of BMI reflecting dyslipidemia in boys.

The ROC curve reflecting dyslipidemia (having one abnormal index in lipids profiles) from BMI-SDS in boys was shown in Figure 4. The area under ROC curve (AUC) of BMI-SDS for predicting dyslipidemia was 0.77(95% CI: 0.70-0.83, p = 0.000). The best cut-off value was 1.22 (sensitivity = 88.4%, specificity = 57.7%). The same method was used to find out the point of BMI to predict dyslipidemia and the value was determined to be 21.67 (sensitivity = 90.7%, specificity = 55.4%) (Figure 5).

Odds ratios for BMI-SDS and BMI in relation to the prevalence of abnormal lipid profiles were presented in Table 5. Subjects with BMI-SDS higher than 1.22 had an adjusted OR of 11.97 (95% CI, 2.75-52.07; p < 0.005) for hypertriglyceridemia compared to those with BMI-SDS lower than 1.22 (adjusted for age). All the adjusted ORs in high BMI-SDS and high BMI group for elevated TC, LDL-C and reduced HDL-C level also increased.

**Table 5:** Adjusted ORs and 95% CIs for dyslipidemia according the cutoff value of BMI-SDS at 1.22 and BMI at 21.67 in boys

	**BMI**-**SDS level**		**BMI**	
	≤**1.22**	**>1.22**	**≤21.67**	**>21.67**
	**(Reference)**		**(Reference)**	
High TC	3 (1.69%)	21 (13.64%)^a^***	2 (1.18%)	22 (12.79%)^a§§§^
Prevalencen (%)				
Adjusted OR	1	8.27 (2.42–28.30)^b^**	1	12.78 (2.95–55.40)^b§§^
High TG	2 (1.13%)	20 (12.20%)^a^***	1 (0.59%)	21 (12.21%)^a§§§^
Prevalence n (%)				
Adjusted OR	1	11.97 (2.75–52.07)^b^**	1	23.82 (3.16–179.39)^b§§^
High LDL-C	2 (1.13%)	19 (11.59%)^a^***	1 (0.59%)	20 (11.63%) ^a§§§^
prevalence n (%)				
adjusted OR	1	11.28 (2.59–49.24)^b^**	1	22.56 (2.99–170.23) ^b§§^
Low HDL-C	1 (0.56%)	12 (7.32%)^a^**	1 (0.59%)	12 (6.78%)^a§§^
prevalence n (%)				
Adjusted OR	1	14.70 (1.88–114.85)^b^*	1	12.20 (1.57–94.92)^b§^
Dyslipidemia	5 (2.82%)	38 (23.17%) ^a***^	4 (2.37%)	39 (22.67%) ^a§§§^
Prevalence n (%)				
Adjusted OR	1	10.27 (3.93–26.84)^b***^	1	12.30 (4.28–35.34)^b§§§^

*OR* Odds ratio, *CI* Confidence interval, *BMI-SDS* Body mass index standard deviation score, ^a^, chi-square test; ^b^, logistic regression analysis adjusted for age.

Differences between groups with BMI-SDS higher and lower than 1.22: **p < 0.005, ***p < 0.001.

Differences between groups with BMI higher and lower than 21.67: ^§^p < 0.05, ^§§^p < 0.005, ^§§§^p < 0.001.

The AUCs of BMI-SDS and BMI in girls for predicting dyslipidemia were close to 0.5 with p values higher than 0.05. Thus the cut-off point in girls couldn’t be determined.

## Discussion

The results of our study showed that obese and even overweight children had dyslipidemia (high TC, TG, LDL-C, insulin and low HDL-C), especially in boys. Thus the reference of Chinese BMI cut-off for overweight and obese can effectively reflect the risk factors of cardiovascular diseases, relatively poor in girls. The concentrations of LDL-C and TG in normal, overweight and obese youths were slightly lower than the results of a study which took WHO cut-off values, while the level of HDL-C was slightly higher [2]. Compared to a survey carried out in Austria youths [7], the average levels of LDL-C in normal weight children were lower, and TC, HDL-C levels were a little higher. The prevalence of high TG, high LDL-C, low HDL-C in the whole population were lower than the results of survey done in USA although there were more normal weight adolescents in their study population (2008 normal weight, 514 overweight, 603 obese) [23] (5.20% *vs*. 8.6%, 6.13% *vs*. 7.5%, 2.97% *vs*. 6.6% respectively). It may due to the racial difference, different dietary pattern and age coverage.

The concentration of TC in our study was consistent with the results of screening study performed among children and adolescents in America [24]. While the fasting glucose levels among normal, overweight and obese children had no statistically differences, the levels of insulin, peptide C and HOMA-IR of overweight and obese children were significantly higher than the normal weight subjects. It may indicate the body’s compensation to control the glucose concentration at the price of hyper secretion of insulin. This finding also matches the results of de Onis’s study [2].

The proportion of children with hypertriglyceridemia and low HDL-C in obese group were about 10.70% and 6.43%, which were lower than 23% and 23.2% reported by de Onis [2]. It may due to the severity of obesity as the mean BMI-SDS of obesity youths in de Onis’s study was 2.73, much higher than 2.46 in our study. The prevalence of abnormal lipid profiles in this total population was also lower than the results of an European research [25]. Besides the severity of obesity differed, the differences of life pattern and race may affected.

The best cut-off of BMI-SDS to dyslipidemia in boys was 1.22, which fell in the range between the values of diagnose of overweight and obesity. This value covers not only obese children but also about two-thirds overweight ones, which is in line with the aggressive stance on lipids screening among overweight and obesity youths recommend by AAP [13]. The points made by Skinner and Maryam Barzin showed that dyslipidemia happened even children were not overweight. The findings of our study seemed to be a little bit high, which mean dyslipidemia happen while children were already overweight. It may due to our relatively small sample or ethnic difference and may request further studies.

Compared to the cutoff points of dyslipidemia or high TG level, the predictive cut-off value of BMI-SDS for low HDL in boys was 2.19, which was much higher than those for high TG and LDL. It also meant something. It was compliance with the research carried out in Italian youths [26]. In 1080 obese children, the average HDL level was 47 mg/dl and even in children with metabolic syndrome the mean level was 37.8 mg/dl, slightly lower than 40 mg/dl. Although the level of HDL significantly differed among healthy BMI, overweight and obese group in this study, the mean level of HDL of obese children was 1.32 mmol/L, even higher than the definition of low HDL by IDF criteria. So we guess that HDL level may be not a sensitive index for diagnose of metabolic syndrome in Chinese youths.

Interestingly, the glycolipid profiles in girls with different BMI did not differ significantly. This phenomenon was not reported by other researchers. One reason was some study did not analyze the gender differences [14]. Although the author offered the data of boys and girls, the differences between genders were not analyzed [6]. The potential cause of this result can be the puberty development during our study age coverage or the small sample of overweight and obese girls.

Though the cut-off value of BMI-SDS and BMI for predicting dyslipidemia in boys was calculated in this study, it should be careful to be applied into practice. The population sample was located in Shanghai urban area, it may not be generalized to other population. The sample size of our study was relatively small, larger studies are needed to confirm or refute our findings.

## Conclusions

The results of this study showed that overweight and obese youths had high levels of TC, TG, LDL, low HDL level and reduced insulin sensitivity. BMI-SDS can predict dyslipidemia at the value of 1.22. Therefore, besides the attention to obese youths, more focus should be casted on overweight ones.

## Abbreviations

BMI-SDS: Body mass index standard deviation score; LDL-C: Low-density lipoprotein cholesterol; HDL-C: High density lipoprotein cholesterol; TG: Triglyceride; TC: Total cholesterol; CVD: Cardiovascular diseases; AAP: American Academy of Pediatrics; NAFLD: Nonalcoholic fatty liver disease; CV: Coefficients of variation; HbA1C%: Hemoglobin A1c; C-P: Peptide C; HOMA-IR: Homeostasis model assessment of insulin resistance; OR: Odds ratio; CI: Confidence interval; ROC: Receiver operating characteristic; AUC: Area under the ROC curve.

## Competing interests

The authors declare that they have no competing interests.

## Authors’ contributions

YMP and ZYZ were responsible for the initial conception and design of the study. CDG, QLW, ZC, DZ carried out the survey.CDG contributed to the statistical analysis and interpretation data and wrote the first draft of the paper. All authors contributed to the critically revision of the article and approved the final published version to be published.
